# Nutritional factors influencing microbiota-mediated colonization resistance of the oral cavity: A literature review

**DOI:** 10.3389/fnut.2022.1029324

**Published:** 2022-10-20

**Authors:** Nuraly S. Akimbekov, Ilya Digel, Adil Y. Yerezhepov, Raiymbek S. Shardarbek, Xia Wu, Jian Zha

**Affiliations:** ^1^Department of Biotechnology, Al-Farabi Kazakh National University, Almaty, Kazakhstan; ^2^Institute for Bioengineering, FH Aachen University of Applied Sciences, Jülich, Germany; ^3^Department of Internal Diseases, Kazakh National Medical University Named After S.D. Asfendiyarov, Almaty, Kazakhstan; ^4^School of Food and Biological Engineering, Shaanxi University of Science and Technology, Xi’an, China

**Keywords:** oral microbiota, biofilm, colonization resistance, nutrition, microbial composition, competition, synergism

## Abstract

The oral cavity is a key biocenosis for many distinct microbial communities that interact with both the external environment and internal body systems. The oral microbiota is a vital part of the human microbiome. It has been developed through mutual interactions among the environment, host physiological state, and microbial community composition. Indigenious microbiota of the oral cavity is one of the factors that prevent adhesion and invasion of pathogens on the mucous membrane, i.e., the development of the infectious process and thereby participating in the implementation of one of the mechanisms of local immunity–colonization resistance. The balance between bacterial symbiosis, microbial virulence, and host resistance ensures the integrity of the oral cavity. In this review we have tried to address how nutritional factors influence integrity of the oral indigenous microbiota and its involvement in colonization resistance.

## Introduction

The oral cavity is considered a unique ecological system, creating favorable conditions for the vital activity of manifold commensal microorganisms that may reside either as planktonic cells or inhabit the biofilms (1). These microbial communities contribute to oral and systemic health by maintaining homeostasis and modulating the immune system (2). The oral cavity becomes colonized with a microbiota, the composition and characteristics of which reflect the local aspects, including potential nutrients, receptors for adhesion, oxygen levels, microbial competitors/collaborators, and local innate and adaptive immune factors. The presence of certain nutrients can lead to a defined spatial architecture within the oral biofilms and contribute to colonization resistance.

Colonization resistance of the oral cavity is a complex, multifaceted phenomenon and characterizes the ability of the resident microbial community to oppose the invasion by exogenous microorganisms. The colonization level, in general, depends on how well the host oral cavity is suitable for growth and on the physiological requirements of the microorganisms. Several principal factors known as “colonization barriers” control the microbial background in direct or indirect pathways (3, 4). Qualitative and quantitative changes in microbiome accompanying the oral cavity’s diseases have been characterized and studied in sufficient detail over the past decade (5, 6).

Especially prominent is the “mucous barrier,” which consists of mechanical, humoral, and other factors protecting the mucous membrane from the colonization of harmful microorganisms (7). In general, the mucous membranes of the lips, palate, cheeks, tongue, gums, teeth, and saliva provide a favorable environment for the growth and reproduction of a wide range of microorganisms. These surfaces are typically densely colonized by complex microbial communities interacting through sophisticated biochemical and biophysical mechanisms (8). Many published studies mention antagonistic activity and adhesiveness of the resident microbiome as the main factors in maintaining intestinal colonization resistance (9). However, the colonization resistance of the oral cavity mediated by nutritional factors remains poorly understood in many aspects. Therefore, the present review furnishes a brief overview the main mechanisms and factors responsible for nutrient-related colonization resistance of the oral microbiota.

## Materials and methods

**FIGURE 1:**
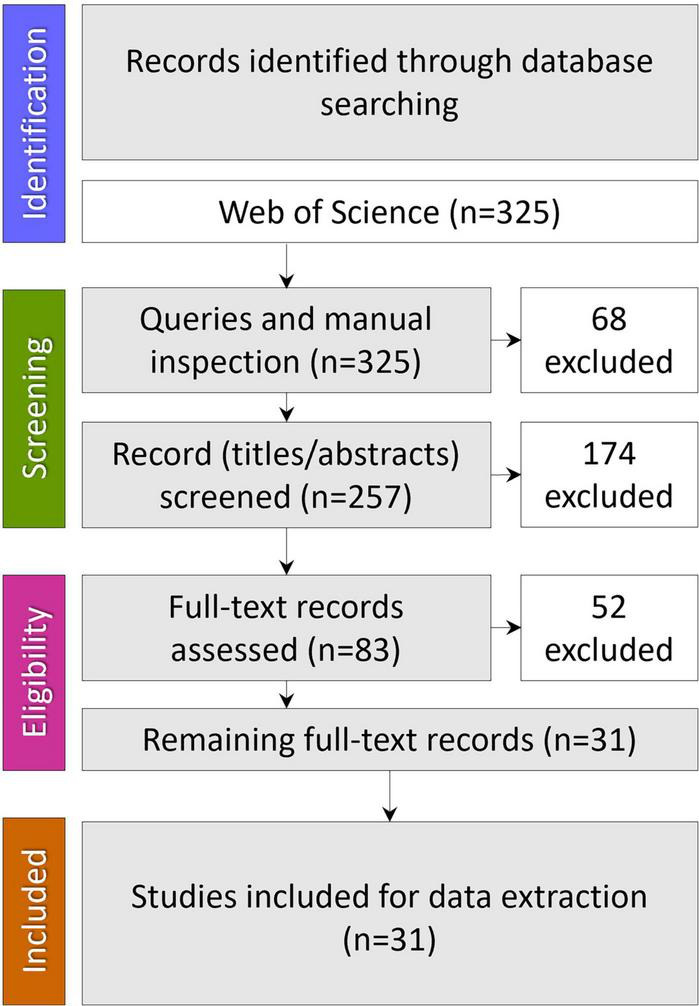
Data identification, screening, eligibility, and inclusion.

A comprehensive literature search was carried out in the online version of the Science Citation Index Expanded (SCI-EXPANDED) from the Web of Science (WoS) database. WoS was chosen as it covers multidisciplinary areas being the oldest citation database. No time restrictions were placed on these searches, and only articles published in English were retrieved. The date when all searches were last performed was September 23, 2022. The search strategy combined three search strings: #1 “oral microbiome” OR “oral microbiota” OR “oral microbiocenosis” OR “oral microbial communities”; #2 “colonization resistance” OR “bacterial interference”; #3 “nutrition” OR “diet” and combining these by “AND” to obtain only the intersection. Results were imported into a bibliographic referencing tool (EndNote 20) and assessed for relevance and quality, removing articles that have no relation to the review topic. Finally, the query results were manually checked before excluding duplicates (Figure 1).

## Results

Our set of queries identified three hundred twenty-five records; after manual inspection and excluding the duplicates, 257 remained. Of these, 174 articles were excluded at the title/abstract level and 52 at the full-text assessment level. Thirty-one were found to meet inclusion criteria to describe the nutritional factors influencing microbiota-mediated colonization resistance of the oral cavity and were used in the analysis.

**FIGURE 2:**
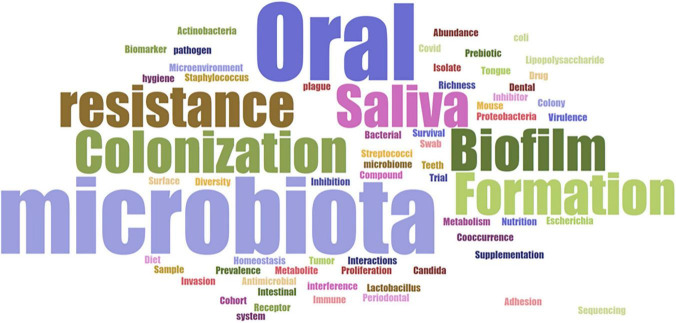
Word cloud based on the main keywords related to the review topic.

The evaluation of the keywords in the included studies is valuable to provide a detailed picture of the review topic, reflecting the research hotspots in the current discipline. Here, the publication keyword analysis to word cloud visualization (Biblioshiny app from the Bibliometrix-R package) revealed that the most common keywords of the thirty-one studies were oral, microbiota, biofilm, formation, saliva, colonization, and resistance (Figure 2). This emphasizes that most studies have focused on biofilm formation and colonization resistance in the oral cavity, as well as saliva’s role in determining the oral microbiota composition.

### Colonization and principal composition of the oral microbiome

The mean total surface area of the mouth is 214.7 ± 12.9 cm^2^ (10), and the mean surface area of the oral mucosa is 196.96 ± 24.20 cm^2^ (11). Each of the anatomical surfaces of the oral cavity, including tooth, gingival sulcus, tongue, hard and soft palates, tonsils, and saliva, is covered with a conglomerate of microorganisms (12, 13). In addition, a large number of microbes are located on the back of the tongue, in the cracks, crevices, and fissures of the tonsils, and in gingival pockets (14).

Intestinal colonization gets its origin from the oral cavity. As the oral cavity is in constant contact with the external environment, it is populated by microorganisms representing a complex biocenosis. In other words, microorganisms making up the microbiocenosis of the oral cavity are intrinsically diverse in their abundance and properties (15). Various microbial taxonomic groups colonize the oral cavity as a kind of ecological niche involving biochemical, immunological, and other interactions with the host. The evolutionary complex and symbiotic communities of microorganisms are therefore specific for a particular area of the mucosal surfaces.

Approximately 99% of all bacteria live together as a biofilm, forming spatially and functionally complex communities (16). Biofilms act as protective shells, making inhabitants more resistant to physical, chemical, and biological factors in comparison with planktonic (free-floating) bacteria (17, 18). In addition to that the biofilm polymers provide adhesion, stabilization, and nutrient flows within the biofilm (19).

Microbial populations in the oral cavity can be divided into resident and transient groups. The resident (indigenous) microbiome includes relatively constant species characteristic of a certain oral biotope and the age of the host. An indigenous microbiome can be divided into core (shared by all host organisms) and variable (due to physiological and biochemical differences between individuals) categories. The transient (exogenous) microbiome consists of non-pathogenic or opportunistic microorganisms that populate the oral cavity for a limited period without causing disease (20). In case of violations or loss of the indigenous microflora, members of the transient can replace the “vacant” niche of a specific biotope that subsequently can contribute to the development of pathology.

The Human Oral Microbiome Database (HOMD^1^) has been created to systematize the bacteria in the human oral cavity, which includes both members of normal microflora and pathogens. HOMD collected 16S rRNA gene sequences from oral prokaryote species into a curated phylogeny-based database. The HOMD contains approximately 772 microbial species, where 70% are culturable, and 57% of which are officially named. Most of the HOMD-listed bacterial species belong to transient microflora since they are not capable of long-term survival under special conditions of the oral cavity. The 16S rDNA profiling of the healthy cavity categorized the inhabitant bacteria into six broad phyla, namely, *Firmicutes, Actinobacteria, Proteobacteria, Fusobacteria, Bacteroidetes*, and *Spirochaetes* constituting 96% of total oral bacteria (21).

*Streptococcus* is the most abundant genus in the oral cavity (8). In the HOMD, the *Streptococcus* genus is represented by 37 species, of which 29 are named, four are not named, and four are lost. The species of *Streptococcus* occupy a specific niche in the oral cavity and thus play a key role in establishing and shaping the oral microbiota (22). *S. gordonii* and *S. oralis* are among the first microorganisms that colonize the oral cavity, followed by cryogenic *S. sanguinis*, *S. mutans*, and *S. sobrinus*, initiating biofilm formation (21). Other pioneer organisms include *Actinomyces* spp. *Granulicatella adiacens*, *Abiotrophia defectiva*, *Gemella* spp., and *Rothia* (23). Diverse molecular forces, including hydrogen bonds, hydrophobic interactions, calcium bridges, van der Waals forces, acid-base interactions, and electrostatic interactions, contribute to the attachment of pioneer bacteria to the salivary acquired pellicle (a layer of proteins and glycoproteins of salivary origin that tightly coat the tooth surface) (24). The early colonizers are predominantly members of the normal microbiota, and just a few are known to be directly responsible for disease development (20). Species of *Streptococcus* initiate numerous cooperative and antagonistic bacterial interactions within the dental microbial community. Thus, mainly streptococci determine and shape the composition of later colonizers in the oral biofilm and greatly impact the health or disease status of the host (22, 25). Polymicrobial colonization and biofilm development have been well-described and depicted by D. Verma et al. (21).

### The role of the normal oral microbiota

Normal microbiota performs protective functions due to indirect antagonism toward pathogenic and opportunistic microorganisms, particularly by preventing colonization of mucous membranes and diminishing the penetration of microbes, microbial toxins, and xenobiotics into the host organism (26, 27). Additionally, the functions of normal oral human microbiota include:

• the regulation of the gas composition of the intestine and other cavities of the host;
• morphokinetic effect;
• the production of enzymes involved in the metabolism of proteins, carbohydrates, lipids, and nucleic acids;
• production of biologically active compounds (vitamins, antibiotics, toxins, hormones, etc.); immunogenic role;
• participation in the recirculation of bile acids, cholesterol, and other macromolecules; mutagenic/antimutagenic role;
• detoxification of exogenous and endogenous substrates and metabolites;
• source of endogenous infection and,

storage of microbial plasmid and chromosomal genes (28–30).

### Nutritional factors influencing the oral microbiota

Saliva is the medium by which the host “supplies” its resident microorganisms with nutrients, including amino acids, proteins, glycoproteins, peptides, and vitamins. In addition, a host-derived nutrient, gingival crevicular fluid (GCF), favors the growth and activity of microorganisms in the oral cavity. In a smaller proportion, the gingival crevice, through GCF secretion, contributes with additional nutrients such as albumin and heme-containing molecules as a source of vital iron (31). Host hormones, such as sex steroid hormones, cholesterol, and catecholamines, delivered through saliva can also be utilized by resident bacteria (32). Many studies suggest that these hormones have the potential to modulate the composition of the oral microbiome (33, 34).

Despite the obvious impact of diet on the oral microbiome, relatively scant information is available regarding this. This can partly be explained by the fact that the primary substrates for oral microbial growth are endogenous nutrients provided by saliva, tissue excludes, GCF, degenerating host cells, or other bacterial metabolites (35).

Studies by Hatakka et al. and Jiang et al. have shown no difference in the growth rates of oral bacteria in the presence or absence of food, indicating no relationship between diet and the composition of oral bacterial communities (36, 37). In contrast, another very recent study by W. G. Wade revealed differences in salivary metabolomic profiles in relation to diet type (omnivorous, ovo-lacto-vegetarian, or vegetarians) (38).

Reduced food intake and fasting periods may affect microbiome-based colonization resistance. The salivary flow and secretion stasis due to dehydration or decreased oral water intake retrograde bacterial migration and colonization (39, 40). Fasting has also been found to be associated with oral cytokine levels caused by resident and transient microbiome (41).

### Oral colonization resistance

**FIGURE 3:**
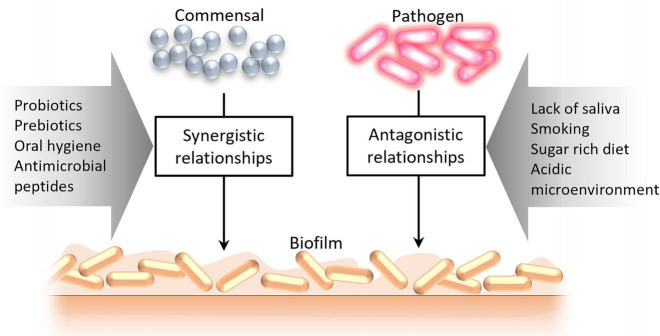
Factors leading to the formation of synergistic and antagonistic relationships between oral microorganisms.

The composition of microbial communities in different biotopes of the oral cavity is determined by environmental and biological factors, giving rise to synergistic or antagonistic relationships (Figure 3). Especially antagonistic relationships between different groups of microbes can be induced by various factors [such as lack of saliva, its bactericidal substances, stimulants (e.g., smoking), increased sugar content, acidic microenvironment] that alter the microbial community structure subsequently impacting colonization resistance (8).

Colonization resistance (also known as bacterial interference) refers to a set of mechanisms providing individual specificity and stability to the microbial community and preventing the host surfaces colonization by pathogens. The term “colonization resistance” was coined by D. van der Waaij (42) who also pointed out that the normal microbiota being a combination of many microbiocenoses characterized by a certain composition and occupying a particular biotope in the human body, plays deciding role in such resistance (43).

In the case of weakened colonization resistance, the fraction of “core” bacteria resident for the surfaces of the human body reduces, while the number and spectrum of potentially pathogenic microorganisms increase. This can lead to their translocation to internal organs and even to the development of purulent-inflammatory processes (44, 45).

Germ-free animals are the primary models showing the pivotal role of resident microflora in colonization resistance and overall health. For example, P. D. Marsh has shown that the absence of resident microbiome has a negative impact on its host, leading to thinning of the intestinal walls, ill-developed villi, poor nutrient absorption, vitamin deficiencies, caecum enlargement, etc., (46). Later, further experimental validations of the role of normal microflora in preventing infections for different microbial models have reported (47–49).

**FIGURE 4:**
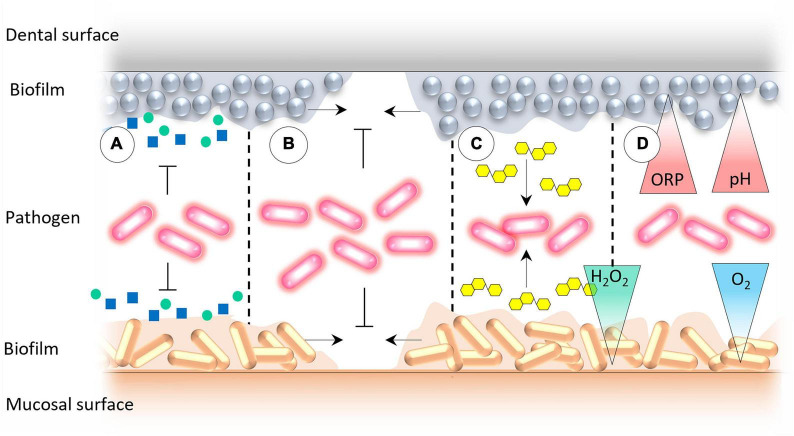
The mechanisms that underpin the oral colonization resistance: **(A)** Competition for essential nutrients and co-factors for microbial growth, **(B)** competition for binding sites for microbial attachment on mucosal and dental surfaces, **(C)** production of antagonistic compounds by the resident oral microbiota, and **(D)** creation of adverse environmental conditions that prevent the growth of exogenous microorganisms.

According to Marsh and Percival (50) the mechanisms mediating colonization resistance can be divided into (a) competition for nutrients, (b) competition for attachment sites, (c)production of antagonistic compounds, and (d) creation of adverse environmental conditions for exogenous microorganisms (Figure 4). Here, we would like to update and address the abovementioned factors in more detail.

#### Nutrient-related factors of colonization resistance

Temperature, pH, atmospheric conditions, salinity, redox potential, sheer and mechanical forces, chemical exposure from hygiene practices, and water of saliva affect the formation of biofilms in the oral cavity (51). However, overall microbial biomass and its composition become considerably controlled by competition for nutritional substances that can be utilized at a low redox potential conditions and in the presence of metabolic inhibitors synthesized by oral microorganisms (52). Salivary amino acids, peptides, GCF, and glycoproteins (such as mucin) are the main limiting nutrient sources for bacteria inhabiting dental plaques. Saliva is the primary nutrient source for bacteria that reside in supragingival biofilm, while GCF provides nutrients for bacteria of the subgingival biofilms (53).

The exact composition of saliva, and therefore the availability of particular nutrients, displays significant interindividual differences as well as high temporary variability (54). In general, resident bacteria outcompete periodontopathogens in the uptake of these nutrients (55–57). However, periodontitis-associated microorganisms destruct tissue through degradation of the extracellular matrix, which leads to additional release of specific nutrients (heme-containing compounds, sources of amino acids, and iron). These nutrients are carried into the gingival crevice through GCF, which favors the atypical growth of asaccharolytic and proteolytic microorganisms with iron-acquisition capacity in the subgingival region (15).

“Food sharing” through bacterial metabolic products also strongly shapes the microbial composition, by encouraging the growth of some species while averting others. For instance, lactic acid, produced by *Streptococcus* and *Actinomyces* in the mouth as a result of carbohydrate fermentation can be utilized by *Veillonella*, allowing for menadione production that is, in turn, important for the growth of *Porphyromonas* and *Prevotella. Fusobacterium* produces fatty acids that are used by *Treponema. Porphyromonas* cal also cooperate with *Treponema* to generate end metabolites that are utilized by *Mogibacterium timidum* (58).

As shown by Van Hoogmoed et al., some conventional oral commensals, such as *S. sanguinis, S. cristatus, S. salivarius, S mitis*, and *A. naeslundii*, decrease the ability of a pathogen *Porphyromonas gingivalis* to adhere to the substrate and retrieve essential nutrients (59). Under optimal conditions, *L. lactis*, a member of the normal oral microbiota, produces nisin, a bacteriocin that mitigates pathogen-mediated oral tumorigenesis (36, 60). Numerous mutualistic nutritional behaviors have also been observed for bacteria growing in saliva as their sole nutrient source (37, 61).

One of the emerging therapeutic approaches could be the introduction of probiotic bacteria which may prevent pathogen colonization in the oral cavity by limiting their adhesion and producing antimicrobials that selectively target disease-associated bacteria (62, 63). For instance, *Streptococcus salivarius* displayed properties compatible with their potential use as probiotics antagonizing *Streptococcus pyogenes* (64–66). However, the main disadvantage here is that the presence of probiotic bacteria can be only transient since they are not indigenous to the oral cavity. Therefore, one more attractive therapeutic strategy could be the nutritional stimulation of indigenous bacteria, promoting oral health.

Indeed, prebiotic substances often induce desirable microbial composition and activity changes, thus delivering local health benefits (67–69). For example, Slomka et al. (69) observed that potential oral prebiotic compounds, such as beta-methyl-D-galactoside and N-acetyl-D-mannosamine selectively stimulate beneficial commensal bacteria of the resident oral microbiome while suppressing the growth of pathogenic bacteria.

Colonization resistance affects the oral ecology in health and disease by preventing or modulating the prevalence of specific microbial groups. Many clinical studies revealed that nutritional compounds are the key factors that alter the oral microbial composition by colonizing microbial biofilms, co-aggregating, and competing with pathogenic bacteria, subsequently reducing/replacing their numbers (Table 1).

**TABLE 1:** Clinical studies to assess the significance of colonization resistance of the oral cavity.

Study type	Patients	Evolved microorganisms	Results	Ref.
Assessment of the microbial prevalence in the oral flora of patients with advanced cancer	Patients with advanced cancer	Yeasts, coliforms, and coagulase-positive staphylococci	A loss of colonization resistance of the oral mucosa during advanced cancer	(70)
The development of new non-invasive differential diagnostic criteria for severity of bronchitis	Children with acute bronchitis	Oral streptococci, *Candida albicans*	Children with bronchitis had significantly lower colonization index and anti-adhesive saliva activity than the control group	(71)
The role of colonization resistance of the oral mucosa in the influence of individual-typological characteristics	Individuals susceptible to caries and inflammatory periodontal diseases	Oral streptococci	Reducing the oral colonization resistance diagnosed in emotionally unstable introverts correlated with their low resistance to periodontal diseases	(72)
The colonization resistance state of the oral mucosa of patients and their dependence on the intensity of the teeth carious	Young patients with different body mass indices	Oral streptococci	In patients with 1st and 2nd degree obesity, in 70% of patients, suppression of oral colonization resistance was observed, compared with patients with average body mass index	(73)
The early diagnosis of the oral cavity’s microecological disorders that assess colonization resistance	Patients with caries and catarrhal gingivitis	Oral streptococci	The development of dental caries and catarrhal gingivitis is accompanied by a decrease in the level of colonization resistance of the oral cavity	(74)
The study of *S. salivarius* to produce a variety of bacteriocin-like inhibitory substances	Healthy patients	*Streptococcus salivarius*	Prevention of streptococcal pharyngitis by anti-*S. pyogenes* inhibitory substances produced by *S. salivarius*	(66)
Application of probiotics Bifidumbacterin in the therapy of periodontal inflammations	Patients with gingivitis and different degrees of periodontitis	*Bifidobacterium bifidum*	Probiotics had a positive effect on the normalization of oral colonization resistance	(75)
*Bacillus subtilis*, as an effective probiotic for prevention of periodontitis	Patients with periodontitis	*Bacillus subtilis*	Mouth rinsing with *B. subtilis* significantly reduced periodontal pathogens	(76)
Reducing the prevalence of oral *Candida* by probiotic-containing cheese	Elderly people	*Lactobacillus rhamnosus, L. rhamnosus, Propionibacterium freudenreichii* ssp. *shermanii*	The probiotic intervention reduced the risk of high *Candida* counts by 75%, and the risk of hyposalivation by 56%	(62)
Assessment of the probiotics to treat gingivitis and evaluation of its influence on plaque	Patients with moderate to severe gingivitis	*Lactobacillus reuteri*	*L. reuteri* was efficacious in reducing gingivitis and plaque through colonization	(77)
Examination of possible effects of *Bifidobacterium* in yogurt on caries-associated microorganisms	Healthy young adults	*Bifidobacterium*	Probiotic bifidobacteria may reduce the levels of selected caries-associated microorganisms in saliva	(78)
Assessment of the beneficial effects *L. rhamnosus* in the oral cavity for long-term caries prevention	Children with the risk of caries	*Lactobacillus rhamnosus*	*L. rhamnosus* was found to reduce the risk of caries significantly, showing antagonism to *Streptococcus mutans*	(79)

#### Other types of colonization resistance

##### Competition for microbial attachment sites on mucosal and dental surfaces

A large number of bacterial species appear to exhibit specific tropism in relation to various anatomical surfaces of the oral cavity. By examining 40 bacterial species, Mager et al. (80) have shown that bacteria that inhabit numerous oral cavity surfaces use very many different receptors and adhesion molecules that define the formation of biofilms (29). Resident oral bacteria form a robust and tight biofilm on the surface of mucous membranes, hindering the adhesion of foreign microorganisms. The most prominent mechanism inhibiting biofilm formation and inducing detachment of extrinsic bacteria from the native biofilm is known as biosurfactant action. Several *in vitro* studies indicated that many bacterial species, especially *Streptococcus*, rely predominantly on this method to prevent foreign colonization of the oral cavity (59, 81, 82). This and other attachment-related strategies are briefly summarized in Table 2.

**TABLE 2:** The mechanisms involved in the competition for attachment sites of colonization resistance.

Mechanisms	Examples	Ref.
Interruption of biofilm formation	*S. cristatus* inhibits the expression of FimA, a gene encoding the major protein subunit of *P. gingivalis* fimbriae	(83–86)
	*S. intermedius* produces arginine deaminase that can repress the expression of FimA and Mfa1 (minor fimbria) in *P. gingivalis*	(87)
Detachment of microorganisms from the biofilm	The transcriptional regulator Nrg1p controls *Candida albicans* biofilm dispersion	(88)
	Modification of the protein composition of the binding site, which is necessary for adhesion of *S. mutans*	(89)
Production of biosurfactants that prevent adhesion	A biosurfactant generated by S. *mitis* decreases the adhesion of *S. mutans* and several periodontopathogens.	(90)

##### Production of antagonistic compounds (inhibitory metabolites)

End products of metabolism of resident microflora are also used for effective protection against extraneous colonization. The antagonism of microorganisms that make up the normal microbiota concerning potentially pathogenic bacteria is due to the production of bacteriocins, lysozyme, and other substances (Table 3).

**TABLE 3:** Production of inhibitory factors by the resident oral microflora that contribute to “colonization resistance”.

Antagonistic agent	Produced by	Against	Ref.
Mutacin, nisin, etc., (lantibiotics and non-lantibiotics)	*S. mutans, Lactococcus lactis*	Gram-positive bacteria, in particular, other streptococci	(91–95)
Sanguicin	*S. sanguinis*	*S. agalactiae* and *S. uberis*	(96)
Salivaricin	*S. salivarius*	a range of streptococci	(96–98)
Reuterin	*Lactobacillus reuteri*	many members of Gram-positive and Gram-negative bacteria	(99)
A bacteriocin	*Lactobacillus paracasei*	*P. gingivalis, Prevotella intermedia, Tannerella forsythensis, S. salivarius, and S. sanguinis*	(100, 101)
Nigrescin	*Prevotella nigrescens*	*P. gingivalis, T. forsythia*, and *Actinomyces* species	(102)
A bacteriocin	*Fusobacterium nucleatum*	a wide range of Gram-negative and Gram-positive bacteria	(103, 104)
Hydrogen peroxide	*S. gordonii*	*S. mutans*	(92, 105)
	*S. sanguinis*	a range of Gram-positive species	(106–112)
	*S. saprophyticus, S. infantis, and S. sanguinis*	non-oral *Escherichia coli*	(113)
	*S. oligofermentans*	*S. mutans*	(114)
Lytic phages	numerous species	numerous species	(115–117)
Nitrite	*S. parasanguinis, S. sanguinis, S. gordonii*	*P. aeruginosa*	(118)

Various bacteriophages represent very abundant and interesting group of oral antimicrobial agents. Oral phages are able to invade many other bacteria besides their putative bacterial hosts. Therefore, phages strongly shape the ecology of oral bacterial communities, accelerate their molecular diversity and help to acquire new gene functions (115–117).

In addition to the metabolic antimicrobials of the microbiota listed in the Table 2, various organic acids should be mentioned, such as short-chain fatty acids (SCFAs), that may act as inhibitory factors (119, 120). Though SCFAs are mainly produced in the intestines, they also contribute to preventing colonization by pathogenic microorganisms in the oral cavity (55).

##### Creation of microenvironments that inhibit the growth of exogenous bacterial species

Here the competition is enabled due to altered environmental conditions, such as pH, oxygen pressure, redox potential, etc., in oral biofilms. The members of *Lactobacillus* and *Streptococcus* are the powerful acid producers, making the local pH drop as low as 4.5, thus dramatically suppressing the growth of all acid-sensitive bacteria (121). Suppression of *S. sanguinis* by mixture of organic acids produced by *S. mutans* has been mentioned in many studies as well (112, 122, 123).

### The factors affecting/influencing colonization patterns

In healthy people, the microbial composition of the oral cavity depends on the physiological and ecological aspects of the host, such as age, nutrition preferences, oral hygiene, anatomical features of the oral cavity, hormonal status, general somatic state, etc., (124). The richness and composition of the oral microbiome are relatively stable due to moisture availability, the constant presence of antimicrobial substances (nisin, diplococcin, acidophilus, lactocidin, lactolin lysozyme, amylases, immunoglobulins A, G, M), organic acids (lactic, acetic, ketoglutaric and succinic) and the state of general cellular and humoral immunity (12).

As described in the previous parts, the colonization resistance is determined by factors of microbial, exogenous, and host origin (125, 126).

#### Microbial factors

Each human individual is characterized by a specific genetically determined spectrum of microorganisms. As we already saw, the normal microflora plays a vital role in the antimicrobial defense system of the oral cavity. The term “normal” indicates a microbial population that colonizes various ecological niches of the healthy oral cavity and takes part in the metabolism of nutrition, protects against highly virulent bacteria by blocking receptors of epithelial cells from adhesion of pathogens, stimulates the immune response, and produces biologically active substances which regulate metabolic processes (127).

#### Exogenous factors

A person’s normal microflora should also be always considered in the context of the whole organism and its environment. The oral cavity is the very beginning of the digestive tract and serves as the “primary portal” for chemical substances and foreign microorganisms. Therefore, numerous external factors affecting the body also affect the microflora. The most pronounced and well-characterized phenomena depend on colonization resistance from smoking status, alcohol consumption, diet (quality and quantity), socioeconomic status, and antibiotic use (125). Smoking is a major environmental factor associated with the pathophysiology of oral diseases. Toxic components in cigarette impact oral microbiota directly or indirectly through oxygen deprivation, immunosuppression, biofilm formation, or other potential mechanisms, leading to loss of colonization resistance (128). Despite different sampling sites, numerous studies have shown the predominance of *Fusobacterium nucleatum* and *F. naviforme* in oral from smokers compared with non-smokers (129). Alcohol consumption may also affect oral microbiota composition affecting functional microbial pathways. Thomas et al. observed the reduced bacterial richness in the oral biofilm of alcohol drinkers (130). Liao et al. found that the genus *Prevotella* and *Moryella* were significantly enriched in drinkers; meanwhile, the genus *Lautropia*, *Haemophilus*, and *Porphyromonas* were depleted significantly (131). Studies indicate that socioeconomic status may alter oral microbiota community structure and higher diversity (132, 133). The oral microbiota is a major reservoir of antibiotic-resistant bacteria; many studies have demonstrated that using amoxicillin, erythromycin, and tetracycline changes oral microbiota composition and enriches bacteria resistant to antibiotics (134, 135).

#### Host factors

Host mechanisms involved in the colonization resistance phenomenon include mucosal desquamation, the antimicrobial effect of secrets, the composition and quantity of mucin, oxygen tension along with the thickness of the biofilm, the pH of the medium, the rate of renewal, maturation and metabolism of mucosal epithelium, innate, and adaptive immune mechanisms, etc., (136). The immune factors, in turn, can involve macrophage activity, lysozyme, lactoferrin, other bactericidal substances of leukocytes, as well as a variety of immunoglobulins, primarily IgA, which prevent microbial adhesion and thus promote the removal of extraneous microorganisms to the external environment (137). The antibacterial potential of saliva on the one side and the number of microorganisms in the oral cavity on the other side exist in dynamic balance. Any infringement of the former leads to disturbances of the normal microflora and the emergence of pathogenic microorganisms by developing various types of pathology in the oral cavity. However, the main functional properties of the host antimicrobial system of saliva not only include suppression of microflora but also effectively control its qualitative and quantitative composition at a level sufficient to maintain microbiocenosis (31, 138).

### Global health relevance

Dental infection and antibiotic resistance remain important global health concerns with significant morbidities. There are convincing scientific pieces of evidence that impaired oral health potentiates the severity of numerous systemic diseases, such as endocarditis, diabetes mellitus, osteoporosis, and tumors (139–142). Severe microbial oral infection and subsequent inflammation, along with meningitis and endocarditis, are reported to be associated with cerebral infractions among male patients (143).

As mentioned above, microbial populations in the oral cavity are of two major types: the resident and transient microbiome; their delicate balance is essential for normal oral functions. Poor oral hygiene, smoking or chewing tobacco, inadequate nutrition, and overuse of antibiotics, not only can disrupt such homeostatic balance between oral resident and transient microbiome, but can also induce antimicrobial resistance (144, 145). Since normal oral microbiota exerts defensive functions against opportunistic harmful microorganisms, developing an approach to restore normal oral microbiota in infectious and inflammatory diseases would likely reduce the oral burden of diseases. The administration of healthy fecal microbiota to restore colonization resistance and displace multi-drug resistant (MDR) bacteria is already a commonly used therapeutic practice (146–148). Whether a similar approach could be employed to restore normal oral microbiota in oral diseases is an area that requires further experimental, theoretical and ethical validation.

A better understanding of microbiota-mediated colonization resistance of the oral cavity would promote rational dental care, and minimize oral-infection related chronic debilitating pathologies, which is also a global health concern. As frequently mentioned, the mouth is the gateway to total body wellness; consequently, the oral microbiome is likely to influence the overall health of an individual. Therapeutic manipulation of the oral microbiome in a patient, by targeting harmful species, to maintain healthier oral status in a community will further assist in the maintenance of good health and well-being, in general.

## Conclusion

The abundance and composition of the oral microbial communities are characterized by the constancy and integrity of the relationships along with the antagonistic and stimulatory effects between microorganisms and their hosts. Colonization resistance is one of the phenomena of local immunity, which depends on a combination of factors that prevent the adhesion and reproduction of exogenous bacteria on dental and mucous surfaces. A certain role in this belongs to resident microflora, which is a potent inhibitor of pathogens and synergist for commensals of the same ecological niche. The antagonistic effect of normal oral microflora is due to the significant adhesive and colonizing ability of resident microbial species, as well as the production of specific substances that inhibit the growth of transient pathogens. Nutritional factors may also modulate microbiota-mediated colonization resistance. So far, the available evidence to assess the real impact of different nutrients on the colonization resistance of the oral microbiome is still insufficient, and more studies are needed.

## Author contributions

All authors contributed to the planning, writing, scientific content, reviewing, editing of this document, and approved the submitted version.

## Footnotes

1
www.homd.org
